# Algorithmically curated music listening and academic stress recovery in university students: examining algorithmic trust, emotional autonomy, and privacy concerns

**DOI:** 10.3389/fpsyg.2026.1887477

**Published:** 2026-07-08

**Authors:** Xiaoning Zheng, Honglin Wang, Qiancai Liu

**Affiliations:** 1 Sejong University, Seoul, Republic of Korea; 2 Carnegie Mellon University, Pittsburgh, PA, United States; 3 Zhengzhou University, Zhengzhou, Henan, China

**Keywords:** academic stress, algorithmic trust, algorithmically curated music, emotional autonomy, mixed methods, privacy concerns

## Abstract

**Background:**

Academic stress is among the most commonly reported psychological difficulties among university students in China, shaped by intense competition, high family expectations, and a rapidly changing graduate employment landscape. Music listening is one of the most widely used informal coping strategies in this population, and Streaming platforms increasingly shape everyday music listening through algorithmically personalized recommendation systems. However, the psychological mechanisms linking algorithmically curated music listening to stress outcomes remain poorly understood. However, the psychological mechanisms linking algorithmically curated music listening to stress outcomes remain poorly understood.

**Methods:**

A sequential explanatory mixed-methods design was employed. A two-wave survey (T1 and T2, 4 weeks apart) was administered to 312 undergraduate students from three Chinese universities. Participants were classified into two listening-type groups based on their self-reported habitual music engagement: predominantly AI-curated (*n =* 178), predominantly self-selected (*n =* 108), or mixed-mode (*n =* 26; excluded from group-comparison analyses). Validated measures of perceived stress (PSS-10), emotional state (PANAS), algorithmic trust, emotional autonomy, and privacy concerns were collected. Multiple regression analyses examined between-group differences and individual-level predictors. A purposive subsample of 24 participants took part in follow-up semi-structured interviews analysed using reflexive thematic analysis.

**Results:**

Students who habitually listened to algorithmically curated music reported moderately lower perceived stress and negative affect at T2 compared to self-selecting listeners, though associations were small in magnitude. Algorithmic trust was the strongest predictor of lower stress (*β* = −0.31, *p* = 0.001). Emotional autonomy showed an unexpected positive association with stress (*β* = 0.19, *p* = 0.027). Privacy concerns were marginally associated with elevated negative affect. Qualitative analysis yielded four themes: low-effort relief, trust as accumulated experience, the double edge of autonomy, and privacy as background noise.

**Conclusion:**

Associations between algorithmically curated music listening and stress recovery depend substantially on the quality of the user–algorithm relationship. Algorithmic trust is a key correlate of favourable outcomes. The design of digital wellbeing tools in higher education should prioritise transparency and user control.

## 1 Introduction

University life in China has never been straightforward, and the pressures students face show little sign of easing. Competitive admissions, demanding curricula, performance-oriented assessment cultures, and an increasingly uncertain graduate job market combine to create a sustained psychological burden that differs in important ways from the stressors described in much of the Western higher education literature (Liu et al., 2019; Zhou and Yao, 2020). National survey data collected before and after the COVID-19 pandemic consistently show that rates of self-reported stress, anxiety, and depressive symptoms among Chinese undergraduates are high by international standards, and formal help-seeking remains inhibited by stigma and limited counselling capacity (Wang et al., 2018).

Music listening is one of the most widely used everyday emotion-regulation strategies among young adults (Saarikallio, 2011). It is readily accessible, socially acceptable, and flexible enough to be used while studying, commuting, or resting. For Chinese university students specifically, music listening ranks among the top three self-reported stress-relief activities, with music widely recognised as serving key emotional functions in young people’s daily coping repertoire (Hargreaves and North, 1999). The technology through which most young people now access music has, however, changed fundamentally. In this article, we use the term “algorithmically curated music listening” to refer to music selected, ranked, or prioritized by platform recommendation systems on the basis of users’ listening histories, behavioural patterns, and platform-level personalization signals. Although some contemporary recommender systems may incorporate machine-learning or AI-based techniques, the present study did not ask participants about specific AI-branded features, such as AI DJ functions or generative music agents. We therefore use “algorithmic recommendation” as the primary analytical term, while reserving “AI” for broader discussions of AI-assisted digital wellbeing technologies. This distinction is important because the present study examines users’ everyday experience of delegating music choice to platform recommendation systems rather than their interaction with a clearly identifiable artificial intelligence agent. Streaming platforms equipped with algorithmic recommendation systems—notably NetEase Cloud Music and QQ Music in the Chinese context—have made algorithmically curated listening the default experience for the majority of users. Rather than actively constructing their own playlists, many students encounter music selected on their behalf by systems they may understand only vaguely, if at all.

This shift creates a set of underexplored questions. Does algorithmically curated music actually help with stress recovery, and if so, does it do so more effectively than music the listener has chosen themselves? Beyond simple efficacy, which psychological characteristics of the listener shape how much they benefit from algorithmically curated music? Three constructs appear particularly relevant. Algorithmic trust—the degree to which a user regards the recommendation system as competent and reliable—is likely to influence how openly students engage with algorithmically generated playlists (Logg et al., 2019). Emotional autonomy—the felt sense of control over one’s own emotional life and coping choices—may moderate whether algorithmic delegation feels supportive or intrusive (Deci and Ryan, 2000). Privacy concerns—awareness of and discomfort with data collection practices—may act as a friction point that shapes engagement with personalised features (Norberg et al., 2007). Despite growing scholarly interest in AI-assisted mental health tools, the intersection of these three constructs with music-based stress recovery has not, to our knowledge, been examined empirically.

The present study addresses this gap through an observational mixed-methods design combining a two-wave survey with follow-up qualitative interviews. Three research questions guide the work:

*RQ1*: Are students who habitually listen to algorithmically curated music associated with lower levels of perceived stress and negative affect over a four-week observation period, compared to those who predominantly self-select their music?

*RQ2*: To what extent do algorithmic trust, emotional autonomy, and privacy concerns predict stress and emotional state outcomes among students who habitually use algorithmically curated music listening?

*RQ3*: How do Chinese university students interpret their experiences with algorithmic music recommendations in the context of managing academic stress?

The remainder of the paper proceeds as follows. Section 2 reviews the theoretical and empirical background. Section 3 describes the study design. Section 4 presents results. Section 5 discusses findings, and Section 6 concludes.

## 2 Literature review

### 2.1 Music listening, algorithmic platforms, and stress/mood management

The relationship between music and psychological stress has been studied from multiple angles, ranging from neurobiological accounts of cortisol suppression and autonomic modulation to social-psychological frameworks emphasising mood regulation and cognitive reappraisal (Juslin and Sloboda, 2010; Thoma et al., 2013). Experimental work has consistently demonstrated that exposure to calming music reduces self-reported anxiety and, under controlled conditions, lowers physiological stress markers including heart rate and skin conductance (Linnemann et al., 2015). What is less settled is whether the listener’s active agency in selecting music contributes to these effects. Most laboratory studies use researcher-selected music, removing individual preference as a variable but also eliminating the sense of personal control that may itself be part of the stress-relief mechanism.

In naturalistic research the picture is more complicated. Saarikallio (2011) showed that young adults use music flexibly across a range of emotion-regulation goals—not always to calm down, but sometimes to amplify or sustain feelings they find meaningful. A system optimising for acoustic features associated with relaxation may occasionally clash with a user’s implicit coping intentions, particularly if those involve emotional processing rather than suppression. The existing literature on music-based stress interventions has not adequately grappled with this complexity, in part because most studies have used researcher-selected music rather than examining what happens when recommendation systems intervene.

Algorithmic music recommendation systems have received considerable attention from computer science researchers, primarily with regard to accuracy and user satisfaction (Schedl et al., 2018). Work addressing their psychological effects is more limited. Ferraro et al. (2021) examined gender imbalance in music recommender systems and demonstrated that collaborative filtering algorithms reproduce existing dataset biases, raising questions about the diversity of emotional content offered to listeners. Anderson et al. (2020) demonstrated that algorithmic recommendation on large streaming platforms can systematically narrow the diversity of content users encounter over time, with direct implications for the range of emotional experiences available through AI-curated listening. More recent work on AI-assisted emotional support technologies has begun to demonstrate that trust in the recommendation system mediates whether users actually benefit from the service (Lee and See, 2004), but this has not been extended to music specifically.

Taken together, this literature suggests that algorithmically curated music listening should not be understood simply as a technical form of personalization. In everyday streaming environments, recommendation systems participate in users’ mood-management practices by reducing the effort required to choose music, shaping the range of affective content encountered, and influencing how much control users feel they retain over their emotional coping strategies. For this reason, the potential relationship between algorithmically curated listening and stress recovery is likely to depend not only on the music delivered, but also on the user’s relationship with the recommendation system.

### 2.2 Algorithmic trust, emotional autonomy, and privacy concerns

To explain why algorithmically curated music may be experienced as supportive by some students but intrusive or ineffective by others, we focus on three user–platform relationship variables: algorithmic trust, emotional autonomy, and privacy concerns.

Trust in automated systems can be usefully separated into cognitive trust (confidence in competence and accuracy) and affective trust (a global sense of comfort with the system) (Komiak and Benbasat, 2006). Logg et al. (2019) demonstrated that people often prefer algorithmic advice to human advice when both are presented as such—a phenomenon they termed ‘algorithm appreciation.’ High-trust users would be expected to engage more fully with AI-curated playlists and ultimately derive greater stress-relief benefits.

Emotional autonomy—the perceived sense of authorship over one’s emotional responses—has its origins in developmental research on adolescent individuation (Barber, 1996; Steinberg and Silverberg, 1986). From a self-determination theory perspective (Deci and Ryan, 2000), high autonomy is generally associated with more adaptive coping. In the context of AI music, however, emotional autonomy may complicate matters: strongly autonomous individuals may resist algorithmic suggestions because accepting them feels like an abdication of control over one’s emotional environment, even when the suggestions are well-matched to their preferences.

Privacy concerns raise specific questions for AI music platforms, which require detailed behavioural data to function effectively. The ‘privacy paradox’ literature documents a persistent gap between stated concerns and actual information-sharing behaviour (Norberg et al., 2007). Survey data suggest privacy awareness among Chinese youth has increased substantially in recent years (Li et al., 2011), yet the regulatory and cultural context may shape the relationship between concerns and behaviour differently than in Western samples. How these dynamics affect engagement with AI music features remains unexplored.

## 3 Methods

### 3.1 Research design and participants

We employed a sequential explanatory mixed-methods design (Creswell and Plano Clark, 2017), beginning with a quantitative phase (two-wave self-report survey) followed by a qualitative interview phase. This was an observational study; no experimental manipulation or random allocation was performed. Ethical approval was obtained from the Institutional Ethics Committee (IEC) of Sejong University (no. IEC/SJU/SPE/1201, dated 01/12/2025) prior to data collection. All participants provided written informed consent.

**Figure 1:**
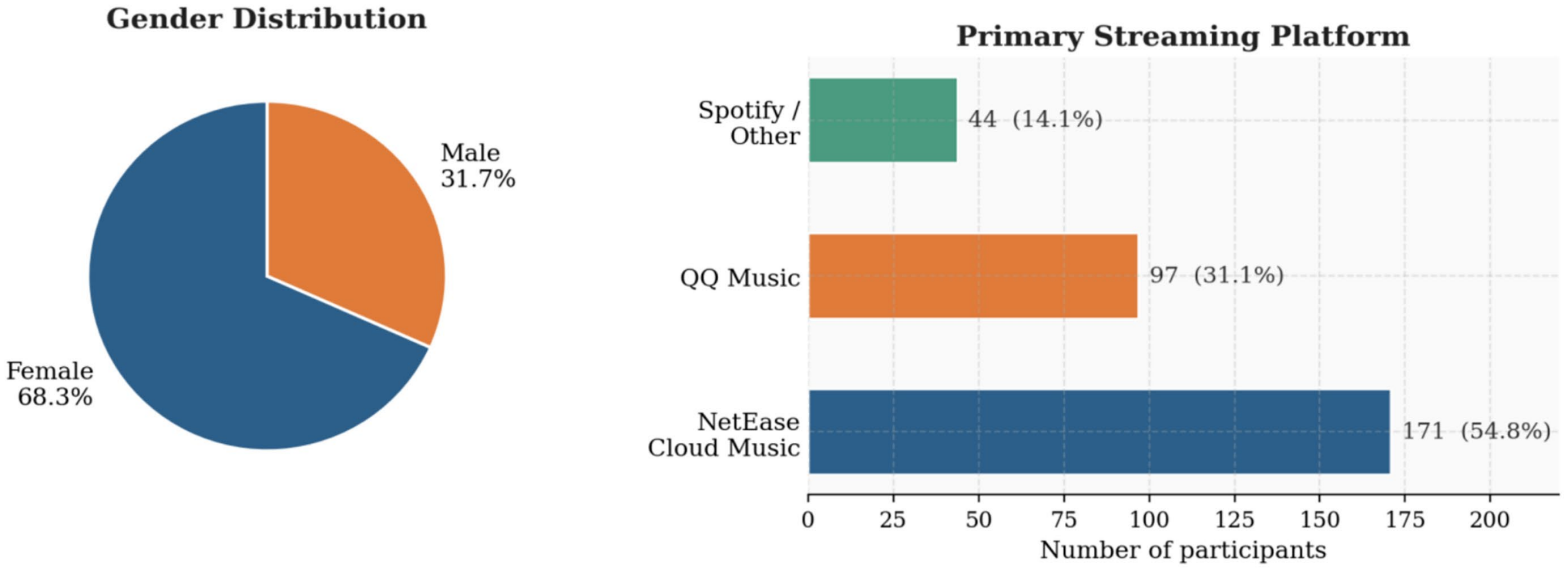
Participant characteristics: gender distribution (left) and primary streaming platform (right) (*N =* 312).

Participants were recruited from three universities in central and eastern China via departmental mailing lists and WeChat group postings between January and March 2025. Eligibility required current undergraduate enrolment, age 18–28, and streaming music use at least three times per week. Of 341 initial expressions of interest, 312 met inclusion criteria and provided complete data after removing incomplete responses (*n =* 12), implausibly fast completions (*n =* 7), and attention-check failures (*n =* 10). Based on their habitual listening behaviour reported at T1, participants were classified into two groups: predominantly AI-curated listeners (*n =* 178; those who reported relying primarily on platform algorithmic playlists), predominantly self-selected listeners (*n =* 108; those who primarily built or chose their own playlists), and mixed-mode listeners (*n =* 26; those who reported using both modes equally). The 26 mixed-mode participants were retained in descriptive analyses but excluded from group-comparison models, yielding a primary analytical sample of 286 participants (AI-curated: *n =* 178; self-selected: *n =* 108) from the overall valid sample of 312. Figure 1 summarises key sample characteristics (full valid sample, *N =* 312). For the qualitative component, a purposive subsample of 24 participants was selected for variation in algorithmic trust scores, gender, and university type; thematic saturation was reached at approximately the nineteenth interview.

### 3.2 Data collection procedure

**Figure 2:**
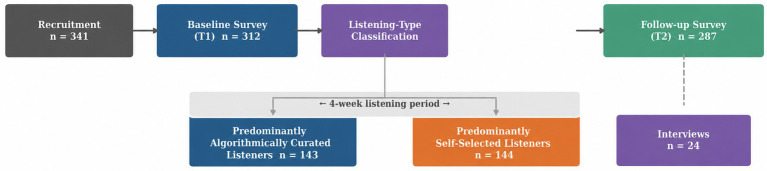
Observational study design and participant flow. Listening type was classified based on participants’ self-reported habitual listening mode at T1; no random allocation or experimental manipulation was performed. The dashed line indicates the qualitative interview component conducted after T2 data collection.

Participants were not randomly assigned to listening conditions; rather, listening type was classified according to their self-reported habitual mode of music engagement at baseline. Figure 2 presents the overall study design and participant flow. At T1 (baseline), participants completed the full survey battery including the PSS-10, PANAS, and all predictor scales, and reported their habitual music listening patterns. Listening type (AI-curated vs. self-selected) was determined from a validated three-item listening habit inventory asking participants to indicate which mode of access they used for the majority of their daily listening. Four weeks later, at T2 (follow-up), participants completed the PSS-10 and PANAS again. Both waves were administered online via Wenjuanxing (a widely used Chinese survey platform). Reminders were sent at weeks two and three. To minimise attrition, participants received a small course-credit incentive for completing both waves. Compliance (i.e., completion of both waves) was 92.0% (*n =* 287 of 312). The three predictor scales were measured at T1 only.

### 3.3 Measures

Listening type was classified via a three-item self-report inventory at T1. Participants indicated their primary mode of daily music access: (1) I mainly let the platform recommend music for me (AI-curated), (2) I mainly build or choose my own playlists (self-selected), or (3) I use both about equally. Participants endorsing option (1) were classified as AI-curated listeners (*n =* 178); those endorsing option (2) as self-selected listeners (*n =* 108); and those endorsing option (3) as mixed-mode listeners (*n =* 26). The 26 mixed-mode participants were retained in descriptive analyses but excluded from the primary group-comparison analyses, leaving 286 participants (178 AI-curated + 108 self-selected) for those models.

Perceived stress was assessed using the Chinese-validated Perceived Stress Scale-10 (PSS-10) (Cohen et al., 1983; Wang et al., 2011), rated on 0–4 frequency scales (*α* = 0.81 at T1; *α* = 0.79 at T2). Emotional state was assessed using the PANAS (Watson et al., 1988), adapted for Chinese samples (Huang et al., 2003) (PA: *α* = 0.84; NA: *α* = 0.78). Algorithmic trust was measured with a six-item scale adapted from Komiak and Benbasat (2004) (*α* = 0.83). Emotional autonomy was assessed using eight items adapted from Barber (1996) psychological autonomy scale, reframed to capture autonomous emotional coping (*α* = 0.76). Privacy concerns were measured with four items adapted from Malhotra et al. (2004) (*α* = 0.79). All predictors used five-point Likert response formats.

### 3.4 Qualitative interviews

Semi-structured interviews (M = 46.2 min, range 38–58 min) were conducted via Tencent Meeting in the 2 weeks following T2. The interview guide covered participants’ general relationship with music and stress, experiences of AI recommendations, sense of control and autonomy, and awareness of data privacy. Interviews were audio-recorded, transcribed verbatim in Mandarin, and translated into English by a bilingual team member (accuracy verified by a second bilingual researcher). Analysis followed Braun and Clarke (2006) reflexive thematic analysis approach, proceeding inductively from the data before mapping themes to quantitative findings.

### 3.5 Analytic strategy

Quantitative analyses were conducted in SPSS 27.0. Independent-samples *t*-tests and Cohen’s d were used to compare T2 PSS-10, PANAS-PA, and PANAS-NA between listening-type groups, with T1 scores entered as covariates in follow-up hierarchical regression models. A series of multiple regression analyses within the AI-curated listener group then examined the unique contributions of algorithmic trust, emotional autonomy, and privacy concerns to T2 outcomes, controlling for T1 baseline. Assumptions of normality, homogeneity of variance, and multicollinearity were examined and met; all variance inflation factors were below 2.3. Because this is an observational study, all group differences are interpreted as associations rather than causal effects. The alpha level was set at 0.05.

## 4 Results

### 4.1 Preliminary analyses and descriptive statistics

Table 1 presents descriptive statistics and zero-order correlations among the main study variables. Baseline PSS-10 scores (M = 19.41, SD = 5.71) were broadly consistent with published norms for Chinese university students (Wang et al., 2011), though the sample skewed toward moderate rather than high stress. The two primary listening-type groups did not differ significantly at T1 on PSS-10 [*t*(284) = 0.63, *p* = 0.53], PANAS-PA [*t*(284) = 0.41, *p* = 0.68], or PANAS-NA [*t*(284) = 1.04, *p* = 0.30], supporting baseline comparability. Algorithmic trust was negatively correlated with T2 PSS-10 (r = −0.22, *p <* 0.01) and PANAS-NA (r = −0.24, *p <* 0.01), and positively correlated with PANAS-PA (r = 0.31, *p <* 0.01). Privacy concerns were negatively associated with algorithmic trust (r = −0.31, *p <* 0.01) (Table 1).

**Table 1:** Descriptive statistics and zero-order correlations among study variables.

Variable	M	SD	1	2	3	4	5	6
1. PSS-10 (T1)	19.41	5.71	–					
2. PSS-10 (T2)	17.86	5.93	0.61**	–				
3. PANAS-PA (T2)	28.14	6.32	−0.29**	−0.34**	–			
4. PANAS-NA (T2)	22.57	5.88	0.44**	0.48**	−0.51**	–		
5. Algorithmic Trust	3.42	0.81	−0.07	−0.22**	0.31**	−0.24**	–	
6. Emotional Autonomy	3.61	0.74	−0.03	−0.18*	0.19*	−0.16*	0.12	–
7. Privacy Concerns	3.28	0.88	0.11	0.09	−0.13	0.17*	−0.31**	−0.08

Descriptive statistics and intercorrelations. Full sample *N =* 312 (AI-curated *n =* 178; self-selected *n =* 108; mixed-mode *n =* 26). Group comparisons used *n =* 286 (AI-curated + self-selected only). *p < 0.05. **p < 0.01 (two-tailed). PSS-10, Perceived Stress Scale; PANAS-PA/NA, Positive/Negative Affect subscales.

### 4.2 Group differences in stress and affect (RQ1)

**Figure 3:**
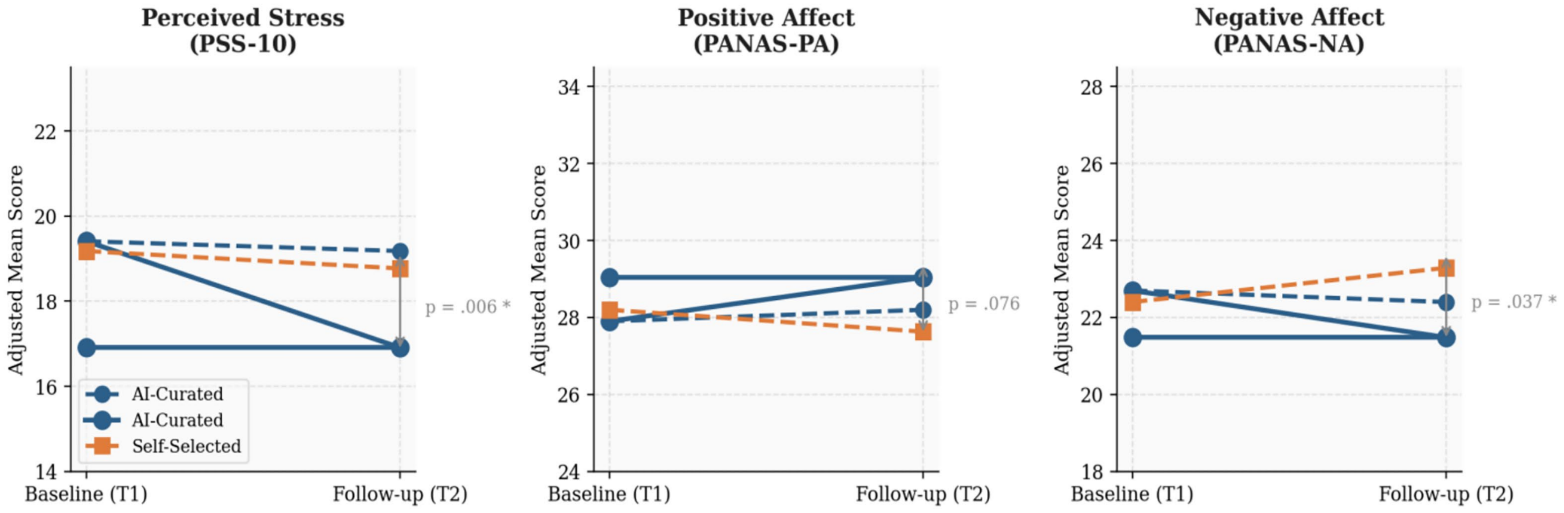
Adjusted T2 outcomes by listening-type group (AI-curated vs. self-selected; primary sample *n =* 286). Error bars represent standard errors (*p < 0.05).

Figure 3 displays the adjusted group means at T1 and T2 across all three outcomes. Hierarchical regression analyses conducted on the primary sample (*n =* 286; Step 1: T1 score; Step 2: listening-type group dummy) revealed that listening type was a significant predictor of T2 PSS-10 (*β* = −0.17, t = 2.80, *p* = 0.006, ΔR^2^ = 0.027), with AI-curated listeners reporting lower adjusted stress scores (M = 16.91, SE = 0.49) than self-selected listeners (M = 18.77, SE = 0.47). The association with PANAS-PA approached but did not reach significance (*β* = 0.13, *p* = 0.076, ΔR^2^ = 0.011). Listening type was marginally associated with lower PANAS-NA (*β* = −0.15, *p* = 0.037, ΔR^2^ = 0.015). This asymmetric pattern—clearer associations with negative affect than positive affect—is discussed in Section 5.

### 4.3 Predictors of stress among AI-curated listeners (RQ2)

Multiple regression within the AI-curated listener group (*n =* 178) examined associations of algorithmic trust, emotional autonomy, and privacy concerns with T2 PSS-10, controlling for T1 stress. The overall model was significant [*F*(4, 173) = 11.24, p = 0.001, R^2^ = 0.245, adjusted R^2^ = 0.224].

**Figure 4:**
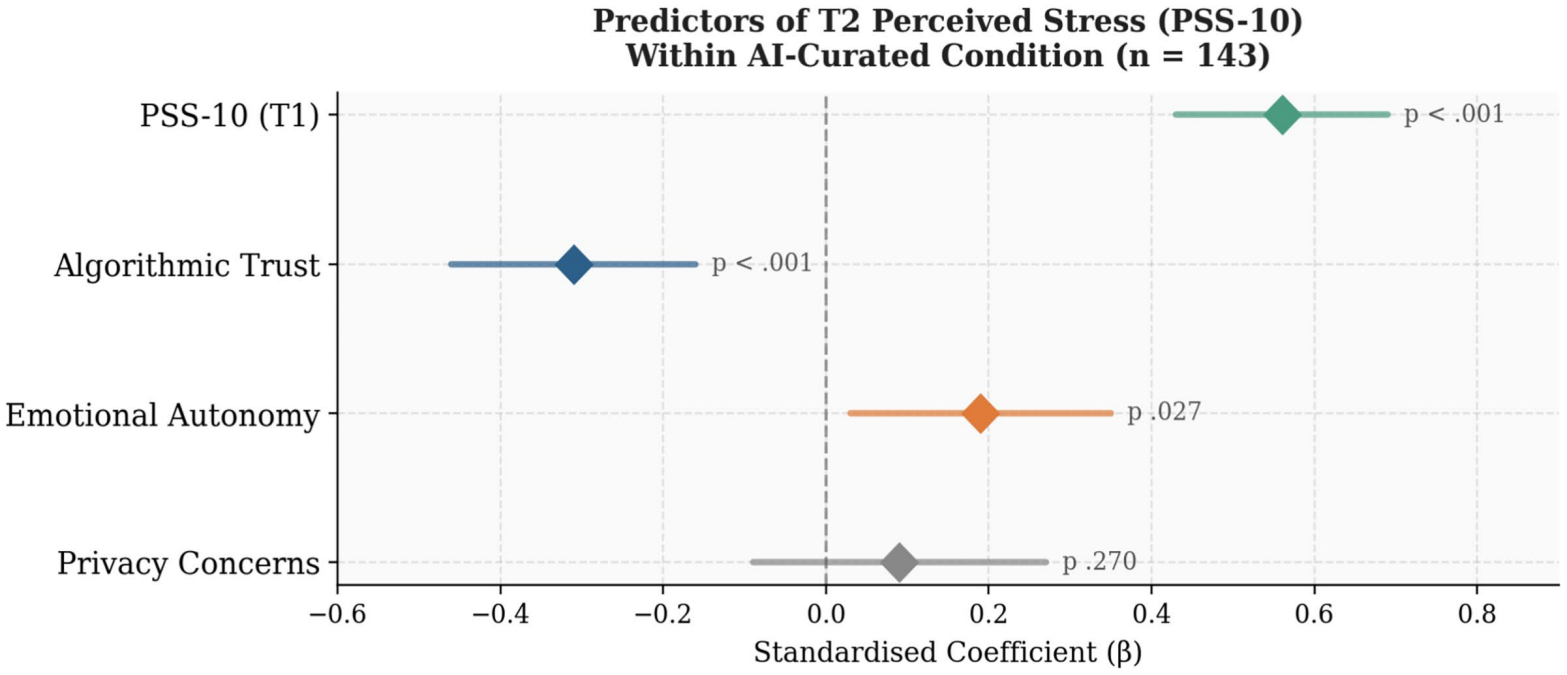
Forest plot of standardised regression coefficients (*β*) predicting T2 PSS-10 within the AI-curated listener group (*n =* 178). Diamonds indicate point estimates; horizontal lines show 95% confidence intervals.

Algorithmic trust was the strongest predictor (*β* = −0.31, *p <* 0.001): higher trust was associated with meaningfully lower follow-up stress, independent of baseline. Emotional autonomy was also significant, but in a counterintuitive direction—higher autonomy predicted higher T2 stress (*β* = 0.19, *p* = 0.027). Privacy concerns did not significantly predict PSS-10 (*β* = 0.09, *p* = 0.270), though they showed a marginal positive association with PANAS-NA (*β* = 0.17, *p* = 0.046) when negative affect was used as the outcome (Figure 4 and Table 2).

**Table 2:** Multiple regression predicting T2 PSS-10 within the AI-curated listener group.

Predictor	B	SE B	*β*	*t*	*p*
PSS-10 (T1)	0.57	0.07	0.56	7.91	< 0.001
Algorithmic Trust	−2.14	0.56	−0.31	−3.84	< 0.001
Emotional Autonomy	1.38	0.62	0.19	2.24	0.027
Privacy Concerns	0.58	0.52	0.09	1.11	0.270

Multiple regression predicting T2 PSS-10 within the AI-curated listener group (*n =* 178). R^2^ = 0.245, adjusted R^2^ = 0.224; *F*(4, 173) = 11.24, *p <* 0.001.

### 4.4 Qualitative findings (RQ3)

Thematic analysis of the 24 interview transcripts yielded four overarching themes. Participant identifiers reflect gender and algorithmic trust tertile (F-HT = female, high trust; M-LT = male, low trust; M-MT = male, mid trust).

*Theme 1: Low-effort relief as its own kind of value*: Many participants described the appeal of AI recommendations in terms of cognitive effortlessness rather than music quality. During high-pressure study periods, the energy required to choose music was experienced as an additional burden:

“When exams are coming I genuinely cannot be bothered to choose. I’ll just let it pick something. It’s not always perfect—sometimes something I’d never have chosen myself—but that’s fine. It’s background, not something I’m actively listening to” (F-HT, Year 3).

*Theme 2: Trust as a product of accumulated experience*: Algorithmic trust, as described by participants, was rarely a general orientation toward recommendation systems but something that had developed—or failed to develop—through repeated interactions with a specific platform:

“I’ve been on NetEase since high school, so it knows me by now. I actually trust it more than I trust myself sometimes—I’ll be in a bad mood and it’ll play something I would not have reached for, and it actually helps” (F-HT, Year 4).

Low-trust participants frequently skipped recommendations or reverted to manual curation when stressed: “It keeps suggesting what’s popular right now, not what I actually like when I’m anxious. After a while I just stopped using the daily recommendations” (M-LT, Year 2).

*Theme 3: The double edge of emotional autonomy*: The qualitative data were particularly useful in illuminating the unexpected positive relationship between emotional autonomy and T2 stress. High-autonomy participants did not simply dismiss AI recommendations; several described recognising that their own choices during stress were not always adaptive:

“I know exactly what I want when I’m anxious. The problem is what I reach for is usually really intense—it feels right but does not actually calm me. The app sometimes pushes me toward something calmer, and I appreciate that intellectually, but I usually skip it anyway” (F-HT, Year 3, high autonomy).

This self-awareness did not easily translate into changed behaviour. The data suggest high emotional autonomy may make algorithmic delegation psychologically uncomfortable even when the individual can see the logic of it.

*Theme 4: Privacy as background noise, not foreground concern*: Privacy concerns were measurable via questionnaire but rarely surfaced in everyday accounts of listening. Most participants displayed practical pragmatism:

“I know they are tracking what I listen to. Everyone knows that. But I do not really think about it when I’m actually using the app. It’s somewhere in the background” (M-MT, Year 2).

A notable exception was one participant who repeatedly deleted and reinstalled his application to reset accumulated algorithmic knowledge, describing recommendations as ‘uncomfortably accurate.’ He held the highest privacy concerns score in the qualitative subsample, illustrating how privacy salience can translate into behavioural disruption that undermines personalisation benefits (Table 3).

**Table 3:** Summary of qualitative themes from semi-structured interviews.

Theme	Core finding	Link to quantitative results
1. Low-effort relief	AI delegation valued for cognitive ease during high-stress periods	Supports modest PSS-10 reduction in AI-curated listener group
2. Trust as accumulated experience	Trust develops through repeated platform interaction, not general AI attitudes	Contextualises strong algorithmic trust → stress recovery path
3. Double edge of autonomy	High-autonomy students recognise but resist helpful AI suggestions	Explains counterintuitive *β* = 0.19 for emotional autonomy
4. Privacy as background noise	Privacy concerns ambient rather than actively disruptive for most users	Clarifies weak direct effect of privacy concerns on PSS-10

Summary of qualitative themes from semi-structured interviews (*n =* 24). Representative excerpts translated from Mandarin.

## 5 Discussion

This study set out to examine whether algorithmically curated music listening supports academic stress recovery among Chinese university students, and to understand the psychological mechanisms that shape individual differences in outcomes. The findings offer a qualified but substantively interesting picture.

The qualitative findings help explain the modest quantitative associations observed in the survey. Rather than suggesting that algorithmically curated music reduces stress simply because the recommended tracks are objectively more effective, the interviews indicate that students often valued recommendation systems because they reduced the effort required to choose music during stressful periods. Similarly, the strong quantitative association between algorithmic trust and lower stress was reflected in participants’ accounts of trust as something accumulated through repeated platform use. The unexpected positive association between emotional autonomy and stress was also clarified by interviews showing that some highly autonomous students resisted algorithmic suggestions even when they recognized that such suggestions might be helpful.

### 5.1 Group differences in observed outcomes

Students who habitually listened to algorithmically curated music reported lower perceived stress and negative affect at four-week follow-up compared to self-selected listeners, though effect sizes were modest (ΔR^2^ = 0.015–0.027). These associations should be interpreted with caution given the observational design: group differences may reflect pre-existing individual characteristics (e.g., openness to algorithmic systems) rather than a direct effect of listening type per se. The asymmetric pattern—clearer associations with negative affect than with positive affect—is consistent with theoretical distinctions between stress relief and wellbeing enhancement as separate psychological processes (MacLeod and Moore, 2000): habitual use of algorithmically curated music may be associated primarily with dampening the aversive dimensions of stress, without reliably elevating positive mood. This suggests AI music tools should be positioned as one component of a broader stress management ecosystem rather than as standalone wellbeing interventions.

The modest effect sizes are consistent with the naturalistic, observational character of the study. Unlike laboratory music interventions that present stimuli in controlled doses (Linnemann et al., 2015), this survey-based design captured habitual behaviour as it already occurs in students’ daily lives, which is both its ecological strength and the source of its interpretive limits. Future research employing longitudinal designs, objective platform listening-log data, and propensity-score matching to account for self-selection would allow stronger causal inference.

### 5.2 Algorithmic trust as a key mechanism

Algorithmic trust was the strongest correlate of lower stress among habitual AI-curated listeners—both statistically and in terms of effect magnitude. This converges with Logg et al. (2019) algorithm appreciation framework and with recent empirical work on trust in AI-assisted health tools (Lee and See, 2004). The qualitative data added a crucial nuance: trust in this context was not a general disposition toward AI but something built through years of platform-specific experience. This has direct design implications. Features that make recommendation logic more transparent, or that provide accurate feedback about why particular tracks were selected, may be more effective at building trust than generic reassurances about AI accuracy. The interview data refine this interpretation by showing that trust was not a general belief that “algorithms are accurate,” but a platform-specific relationship developed through repeated listening experiences. This helps explain why algorithmic trust predicted stress outcomes more strongly than privacy concerns or emotional autonomy: trusted systems were experienced as reliable partners in low-effort mood management, whereas low-trust systems were treated as sources of irrelevant or intrusive suggestions.

### 5.3 The unexpected role of emotional autonomy

The positive association between emotional autonomy and T2 stress within the AI-curated listener group—contrary to initial expectations—is among the study’s most theoretically interesting findings. The qualitative data suggest that high-autonomy students are not indifferent to AI recommendations; rather, they actively resist them even while recognising, intellectually, that the suggestions might be helpful. This is consistent with self-determination theory’s prediction that experiences of external control undermine intrinsic motivation (Deci and Ryan, 2000)—even when the ‘controller’ is a music algorithm and the user is technically free to override it at any moment.

An alternative interpretation is that the relationship between emotional autonomy and stress is moderated by algorithmic trust: high-autonomy, low-trust individuals may report higher stress in naturalistic AI-curated listening contexts, while high-autonomy, high-trust individuals might show no deficit or even benefit. The sample size was insufficient to test this interaction reliably, but it would be a productive focus for future research with larger samples. This finding suggests that personalization and autonomy should not be treated as automatically compatible. In algorithmically curated listening, personalization may reduce the burden of choice while also making some users feel that their emotional coping has been partially delegated to the platform. For students who strongly value emotional self-direction, this delegation may be experienced as uncomfortable even when the recommendations are musically appropriate.

### 5.4 Privacy concerns as background friction

Privacy concerns showed a more muted pattern than anticipated. They did not directly predict PSS-10 outcomes and were only marginally associated with negative affect. The qualitative data contextualise this: for most participants, privacy concerns functioned as ambient awareness rather than active preoccupation—broadly consistent with the privacy paradox (Norberg et al., 2007). The outlier case of the participant who repeatedly reinstalled his application to reset his algorithmic profile illustrates, however, that for a subset of users with high privacy salience, concerns can translate into behavioural disruption that undermines personalisation benefits. Longitudinal studies tracking whether privacy concern effects intensify as students’ data literacy grows would be valuable. The qualitative findings suggest that privacy concerns operated less as an immediate barrier to music listening and more as background friction in the personalization process. Most students were aware that platforms collected listening data, but this awareness became behaviourally relevant only when recommendations felt uncomfortably accurate or overly intrusive.

### 5.5 Limitations

Several limitations should be acknowledged. Most importantly, the observational design precludes causal inference: group differences in stress outcomes may reflect pre-existing characteristics (e.g., openness to algorithms, personality traits) that simultaneously drive both listening preferences and stress levels. Future research using experimental or quasi-experimental designs (e.g., instrumental variable approaches, regression discontinuity) would be needed to establish directionality. The four-week follow-up interval may not capture longer-term dynamics of trust development or habituation effects. The sample was drawn from three institutions and skewed toward moderate stress, and may not represent students at very high-pressure or less urban universities. Listening type was classified via self-report rather than objective platform data, introducing potential misclassification. Exclusion of the 26 mixed-mode listeners from primary group comparisons reduces statistical power and may not fully represent the growing proportion of students who flexibly alternate between algorithmic and self-selected listening. The predictor scales were adaptations of existing instruments rather than purpose-built measures for this context, and formal construct validation in this specific application remains to be established.

## 6 Conclusion

Algorithmically curated music listening is already embedded in the daily lives of most Chinese university students. This observational study provides mixed-methods evidence that habitual algorithmically curated music listening is associated with modestly lower perceived stress and negative affect over a four-week period, and identifies algorithmic trust as the strongest correlate of favourable outcomes among AI-curated listeners. The counterintuitive role of emotional autonomy—associated with worse outcomes in the AI-curated listener group—adds important nuance and points to the importance of preserving users’ felt sense of control when designing AI-based mental health tools.

The study’s core practical message is that the psychological benefits of AI music tools are not automatic or universal. They depend on the quality of the relationship that develops between user and algorithm over time—a relationship shaped by accumulated experience, individual differences in autonomy orientation, and ambient privacy concerns that most users carry without necessarily acting upon. Designing for this relational dimension, rather than treating personalisation as a purely technical optimisation problem, is a productive direction for digital mental health support in higher education.

Future research would benefit from experimental or longitudinal designs that allow causal inference, longer follow-up periods, objective platform listening data, and cross-cultural replication in contexts where the regulatory and cultural relationship to data privacy differs from contemporary China.

## Data Availability

The original contributions presented in the study are included in the article/Supplementary material, further inquiries can be directed to the corresponding author.

## References

[ref1] Anderson A. Maystre L. Anderson I. Mehrotra R. Lalmas M. (2020). Algorithmic effects on the diversity of consumption on spotify. In Proceedings of the web conference 2020 (pp. 2155–2165). 10.1145/3366423.3380281

[ref2] Barber B. K. (1996). Parental psychological control: revisiting a neglected construct. Child Dev. 67, 3296–3319. doi: 10.2307/1131780, 9071782

[ref3] Braun V. Clarke V. (2006). Using thematic analysis in psychology. Qual. Res. Psychol. 3, 77–101. doi: 10.1191/1478088706qp063oa

[ref5] Cohen S. Kamarck T. Mermelstein R. (1983). A global measure of perceived stress. J. Health Soc. Behav. 24, 385–396. doi: 10.2307/2136404, 6668417

[ref6] Creswell J. W. Plano Clark V. L. (2017). Designing and Conducting Mixed Methods Research. 3rd Edn Thousand Oaks, CA: Sage.

[ref8] Deci E. L. Ryan R. M. (2000). The 'what' and 'why' of goal pursuits: human needs and the self-determination of behavior. Psychol. Inq. 11, 227–268. doi: 10.1207/s15327965pli1104_01

[ref9] Ferraro A. Serra X. Bauer C. (2021). “Break the loop: Gender imbalance in music recommenders,” in *Proceedings of the 2021 Conference on Human Information Interaction and Retrieval* (249–254).

[ref10] Hargreaves D. J. North A. C. (1999). The functions of music in everyday life: redefining the social in music psychology. Psychol. Music 27, 71–83. doi: 10.1177/0305735699271007

[ref11] Huang L. Yang T. Ji Z. (2003). Applicability of the positive and negative affect scale in Chinese. Chin. Ment. Health J. 17, 54–56.

[ref12] Juslin P. N. Sloboda J. A. (2010). Handbook of Music and Emotion: Theory, Research, Applications. Oxford: Oxford University Press.

[ref14] Komiak S. Y. X. Benbasat I. (2004). Understanding customer trust in agent-mediated electronic commerce, web-mediated electronic commerce, and traditional commerce. Inf. Technol. Manag. 5, 181–207. doi: 10.1023/b:item.0000008081.55563.d4

[ref15] Komiak S. Y. X. Benbasat I. (2006). The effects of personalization and familiarity on trust and adoption of recommendation agents. MIS Q. 30, 941–960.

[ref16] Lee J. D. See K. A. (2004). Trust in automation: designing for appropriate reliance. Hum. Factors 46, 50–80. doi: 10.1518/hfes.46.1.50.30392, 15151155

[ref17] Li H. Sarathy R. Xu H. (2011). Understanding situational online information disclosure as a privacy calculus. J. Comput. Inf. Syst. 51, 62–71. doi: 10.1080/08874417.2010.11645450

[ref18] Linnemann A. Ditzen B. Strahler J. Doerr J. M. Nater U. M. (2015). Music listening as a means of stress reduction in daily life. Psychoneuroendocrinology 60, 82–90. doi: 10.1016/j.psyneuen.2015.06.008, 26142566

[ref20] Liu X. Ping S. Gao W. (2019). Changes in undergraduate students' psychological wellbeing as they experience university life. Int. J. Environ. Res. Public Health 16:2864. doi: 10.3390/ijerph16162864, 31405114 PMC6719208

[ref21] Logg J. M. Minson J. A. Moore D. A. (2019). Algorithm appreciation: people prefer algorithmic to human judgment. Organ. Behav. Hum. Decis. Process. 151, 90–103. doi: 10.1016/j.obhdp.2018.12.005

[ref22] MacLeod A. K. Moore R. (2000). Positive thinking revisited: positive cognitions, well-being and mental health. Clin. Psychol. Psychother. 7, 1–10.

[ref23] Malhotra N. K. Kim S. S. Agarwal J. (2004). Internet users' information privacy concerns (IUIPC). Inf. Syst. Res. 15, 336–355. doi: 10.1287/isre.1040.0032, 19642375

[ref24] Norberg P. A. Horne D. R. Horne D. A. (2007). The privacy paradox: personal information disclosure intentions versus behaviors. J. Consum. Aff. 41, 100–126. doi: 10.1111/j.1745-6606.2006.00070.x

[ref25] Saarikallio S. (2011). Music as emotional self-regulation throughout adulthood. Psychol. Music 39, 307–327. doi: 10.1177/0305735610374894

[ref26] Schedl M. Zamani H. Chen C. W. Deldjoo Y. Elahi M. (2018). Current challenges and visions in music recommender systems research. Int. J. Multimed. Inf. Retr. 7, 95–116. doi: 10.1007/s13735-018-0154-2

[ref27] Steinberg L. Silverberg S. B. (1986). The vicissitudes of autonomy in early adolescence. Child Dev. 57, 841–851. doi: 10.2307/1130361, 3757604

[ref28] Thoma M. V. La Marca R. Brönnimann R. Finkel L. Ehlert U. Nater U. M. (2013). The effect of music on the human stress response. PLoS One 8:e70156. doi: 10.1371/journal.pone.0070156, 23940541 PMC3734071

[ref30] Wang Z. Chen H. Sun X. (2011). Reliability and validity of the PSS-10 in Chinese university students. Chin. J. Clin. Psychol. 19, 16–18.

[ref31] Wang J. Mann F. Lloyd-Evans B. Ma R. Johnson S. (2018). Associations between loneliness and perceived social support and outcomes of mental health problems: a systematic review. BMC Psychiatry 18:156. doi: 10.1186/s12888-018-1736-5, 29843662 PMC5975705

[ref32] Watson D. Clark L. A. Tellegen A. (1988). Development and validation of brief measures of positive and negative affect: the PANAS scales. J. Pers. Soc. Psychol. 54, 1063–1070. doi: 10.1037/0022-3514.54.6.1063, 3397865

[ref33] Zhou X. Yao B. (2020). Social support and acute stress symptoms (ASSs) during the COVID-19 outbreak: deciphering the roles of psychological needs and sense of control. Soc. Psychiatry Psychiatr. Epidemiol. 11:1779494. doi: 10.1080/20008198.2020.1779494 PMC7534382 33062202

